# Exploitation of *Drosophila* Choriogenesis Process as a Model Cellular System for Assessment of Compound Toxicity: the Phloroglucinol Paradigm

**DOI:** 10.1038/s41598-019-57113-3

**Published:** 2020-01-14

**Authors:** Konstantinos E. Keramaris, Konstantinos Konstantopoulos, Lukas H. Margaritis, Athanassios D. Velentzas, Issidora S. Papassideri, Dimitrios J. Stravopodis

**Affiliations:** 1 Department of Hematology and Bone Marrow Transplantation, Medical School, National and Kapodistrian University of Athens (NKUA), Athens, Greece; 2 Section of Cell Biology and Biophysics, Department of Biology, School of Science, National and Kapodistrian University of Athens (NKUA), Athens, Greece

**Keywords:** Cell biology, Chemical biology

## Abstract

Phloroglucinol (1,3,5 tri-hydroxy-benzene) (PGL), a natural phenolic substance, is a peroxidase inhibitor and has anti-oxidant, anti-diabetic, anti-inflammatory, anti-thrombotic, radio-protective, spasmolytic and anti-cancer activities. PGL, as a medicine, is administered to patients to control the symptoms of irritable bowel syndrome and acute renal colic, in clinical trials. PGL, as a phenolic substance, can cause cytotoxic effects. Administration of PGL up to 300 mg/kg (bw) is well tolerated by animals, while in cell lines its toxicity is developed at concentrations above the dose of 10 μg/ml. Furthermore, it seems that tumor or immortalized cells are more susceptible to the toxic power of PGL, than normal cells. However, studies of its cytotoxic potency, at the cellular level, in complex, differentiated and meta-mitotic biological systems, are still missing. In the present work, we have investigated the toxic activity of PGL in somatic epithelial cells, constituting the follicular compartment of a developing egg-chamber (or, follicle), which directs the choriogenesis (i.e. chorion assembly) process, during late oogenesis of *Drosophila melanogaster*. Our results reveal that treatment of *in vitro* growing *Drosophila* follicles with PGL, at a concentration of 0.2 mM (or, 25.2 μg/ml), does not lead to follicle-cell toxicity, since the protein-synthesis program and developmental pattern of choriogenesis are normally completed. Likewise, the 1 mM dose of PGL was also characterized by lack of toxicity, since the chorionic proteins were physiologically synthesized and the chorion structure appeared unaffected, except for a short developmental delay, being observed. In contrast, concentrations of 10, 20 or 40 mM of PGL unveiled a dose-dependent, increasing, toxic effect, being initiated by interruption of protein synthesis and disassembly of cell-secretory machinery, and, next, followed by fragmentation of the granular endoplasmic reticulum (ER) into vesicles, and formation of autophagic vacuoles. Follicle cells enter into an apoptotic process, with autophagosomes and large vacuoles being formed in the cytoplasm, and nucleus showing protrusions, granular nucleolus and condensed chromatin. PGL, also, proved able to induce disruption of nuclear envelope, activation of nucleus autophagy (nucleophagy) and formation of a syncytium-like pattern being produced by fusion of plasma membranes of two or more individual follicle cells. Altogether, follicle cell-dependent choriogenesis in *Drosophila* has been herein presented as an excellent, powerful and reliable multi-cellular, differentiated, model biological (animal) system for drug-cytotoxicity assessment, with the versatile compound PGL serving as a characteristic paradigm. In conclusion, PGL is a substance that may act beneficially for a variety of pathological conditions and can be safely used for differentiated somatic -epithelial- cells at clinically low concentrations. At relatively high doses, it could potentially induce apoptotic and autophagic cell death, thus being likely exploited as a therapeutic agent against a number of pathologies, including human malignancies.

## Introduction

Phloroglucinol (1,3,5 tri-hydroxy-benzene) (PGL) is a natural phenolic substance, member of the phloroglucinols family, consisting of more than 700 naturally occurring derivatives^1,2^. In many countries, PGL is being used as a medicine to relieve pains and symptoms associated with irritable bowel syndrome, while it is also an anti-spasmodic agent acting on smooth muscle^3–7^. Interestingly, double-blind randomized clinical trials demonstrated that intravenous administration of 40 mg PGL reduces the duration of 1^st^, 2^nd^ and 3^rd^ stage of labor, and has analgesic actions, without toxic effects on mother and fetus^8,9^. PGL has been extensively studied as a substance with anti-diabetic^10–12^, anti-stress^13^, anti-inflammatory^14^, anti-coagulant^15,16^, anti-oxidant^17–22^, radio-protective^23–25^ and anti-cancer^26–32^ activities. Nevertheless, the cytotoxicity of PGL in differentiated, and non-mitotic, somatic cells that are being organized in complex biological systems has not been reported yet.

Thereby, for investigating the PGL toxic effects on functional integrity and structural architecture of multi-cellular, differentiated and non-mitotic settings, we have herein engaged the process of chorion (egg-shell) synthesis (choriogenesis) during *Drosophila melanogaster* late oogenesis, which offers the unique and invaluable advantage of *in vitro* follicle-development completion within a few hours. Chorion is a complex extracellular-protein structure being formed at the final stages of fly-follicle maturation and it consists of multiple successive layers; the wax layer, the -crystalline- inner chorionic zone (ICL), the tri-partite endochorion (organized in floor, pillars and roof) and the fibrous exochorion, externally bordering the vitelline membrane and the oocyte. The complexity of chorion structure requires tight spatio-temporal control of cell function^33,34^. Indicatively, we can report the differentiation of cell sub-populations, the cell migration, the synthesis, modification and secretion of chorionic proteins, and the programmed cell death of follicular epithelium^35–37^.

More than 30 proteins are components of the assembled chorion structure^38,39^. The most quantitatively abundant proteins are the “early” s38 and s36, “middle” s19 and s18, and “late” s16 and s15 ones, whose massive production is mainly regulated by a gene-amplification process^40^. Most importantly, the egg-shell peroxidase (ESP) serves as a major structural and enzymatic component of *Drosophila* chorion. ESP is activated at the end of choriogenesis by endogenous hydrogen peroxide, and creates di- and tri-tyrosine covalent bonds among -chorionic- protein components^41,42^.

Importantly, during the late stages of *Drosophila* oogenesis, ovarian follicle cells are subjected to apoptotic and autophagic cell death programs that allow them to detach from the egg-shell when the mature follicle exits the ovariole^37,43^. Apoptosis is characterized by shrinkage of the cell, condensation of chromatin, fragmentation of nuclear genome, blebbing of plasma membrane and clearance of generated debris by neighboring skillful phagocytes^44,45^. On the other hand, autophagy can be distinguished by vacuolization of the cytoplasm, formation of autophagosomes and lysosome-mediated clearance of the engulfed material^46,47^.

Aim of this study is the investigation of PGL cytotoxic power and the determination of substance concentration that does not adversely affect cellular physiology. The toxicity limit in differentiated, somatic -epithelial- cell (sub-)populations that are being organized in complex and “mitotically inactive” compartments is a very useful and powerful parameter to estimation of the optimum drug-dose administration in a given pathology-treatment protocol, seeking for maximal therapeutic benefits and minimal toxic -side- effects.

## Materials and Methods

### Drosophila melanogaster culturing

The wild-type strain Oregon-G of *Drosophila melanogaster* was cultured in glass tubes under standard conditions (25 °C with a photoperiod of 12 h light and 12 h darkness). Fly food contained 0.8% agar, 2.6% yeast, 6.4% corn-flour, 3.2% sugar, 5.0% tomato-paste and 0.4% propionic acid^48^. The cooked meal was dispensed in the culture vials and 2–3 drops of wet yeast were added in every vial.

Newly emerged flies were transferred in fresh food for 3–4 days before dissection.

### Dissection of flies

Flies of 3–4 days old were anesthetized via their exposure to di-ethyl-ether for 10–15 sec. Females were selected and their ovaries were hand-dissected under a stereo-microscope in cold *Drosophila* Ringer’s solution^49^. Follicles at the developmental stages 11–14 were separated from ovarian tissues, using anatomy micro-needles, according to morphological criteria, described by Margaritis, 1980, and carefully placed into sterilized micro-tissue wells containing Robb’s culture medium^50^.

### *In vitro* development of ovarian follicles

**Figure 1:**
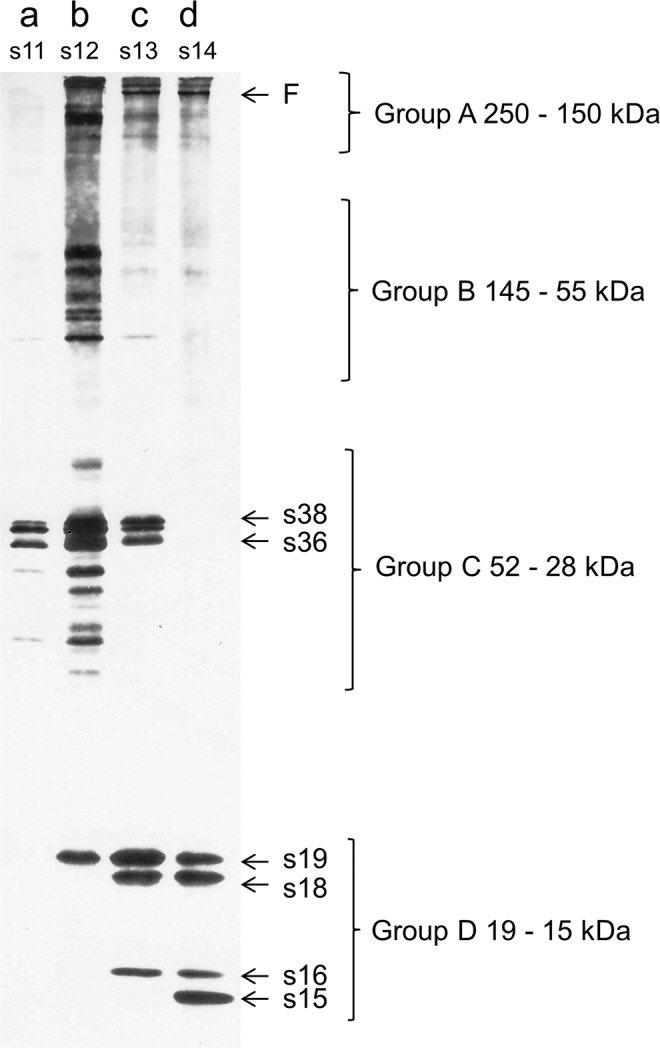
Developmental profiling of chorionic-protein synthesis in the presence of 0.2 mM PGL. SDS-PAGE (10–15% gradient) and autofluorography analysis of the developmentally regulated and stage-specific *D. melanogaster* chorionic proteins, after follicles’ *in vitro* growth in Robb’s nutrient medium in the presence of [^3^H] radio-labeled L-proline and 0.2 mM of PGL concentration. (**a**) stage-11 follicles (s11), 30 min incubation time; (**b**) stage-12 follicles (s12), 120 min incubation time; (**c**) stage-13 follicles (s13), 60 min incubation time; and (**d**) stage-14 follicles (s14), 120 min incubation time. Group A: high-molecular weight chorionic proteins of 250–150 kDa, including the filament-specific F protein (s13 and s14). Group B: chorionic proteins with a molecular-weight range from 145 to 55 kDa, including the s95, s90, s80, s75 and s70 ones previously reported^52,76^. Group C: chorionic proteins with a 52 to 28 kDa molecular-weight range, containing the major “early” chorionic proteins s38 and s36, and the group of proteins being resolved below the s36 one, as similarly described for *D. virilis* species^77^. Group D: the major “middle” and “late” chorionic proteins s19, s18, s16 and s15.

Follicles of the desired developmental stage(s) were cultured in Robb’s medium, which, in the radio-labeling *in vitro* experiments, was supplemented with [^3^Η] radio-active L-proline (1 mCi/ml, Amersham, England, UK). PGL (Merck, Germany) was added in Robb’s medium from stock solution, at a range of 0.2 to 40 mM final concentration. Follicles were carefully separated from ovaries and, next, were transferred to glass-wells containing Robb’s medium, as described by Petri and colleagues^51^. Isolated follicles were allowed to grow *in vitro* for 6 or 12 h. With regard to the effect of 0.2 mM PGL, its incubation time was set for stage 11 at 30 min, for stage 12 at 120 min, for stage 13 at 60 min and for stage 14 at 120 min (Fig. 1)^38,51,52^.

### SDS-PAGE electrophoresis and autofluorography

After their *in vitro* development in radio-active culture medium, follicles were washed with cold Ringer’s and, next, were solubilized in STG (2% SDS, 10% glycerol and 50 mM Tris-HCL, pH 6.8) lysis buffer. Total-protein extracts were analyzed on SDS-PAGE^53^ containing a 10 to 15% linear-gradient of acrylamide, as described by Petri and colleagues^38^. Chorion-protein expression profiles were unveiled by an autofluorography procedure being based on the incorporation of radio-active proline. For the detection of radio-labeled chorionic proteins, slab gels were immersed in a DMSO-PPO solution^54^ and the dried gels were exposed to −80 °C, using XAR-5 Kodak plates^55^. No digital image processing of any kind has been applied to the presented gels.

### Transmission electron microscopy

Dissected follicles, after their *in vitro* growth in non-radio-active Robb’s medium, were, next, fixed by a solution containing 2% formaldehyde and 2% glutaraldehyde in 80 mM sodium cacodylate buffer, followed by 2% osmium tetroxide in ddH_2_O, and were immediately prepared for transmission electron microscopy (TEM), according to the method described by Margaritis and colleagues^34^. TEM preparations (thin resin-section staining with uranyl acetate and lead citrate) were examined using a Philips EM-300 operating at 60 kV.

## Results

### *In vitro* development of *Drosophila* follicles in Robb’s culture medium containing [^3^H] radio-labeled L-proline and 0.2 mM PGL

The *D. melanogaster* chorion is formed during the late stages of choriogenesis, 11–14. The length of each developmental stage was determined as previously described^38,52^. Biochemical analysis of chorionic proteins in *Drosophila* necessitates the inhibition of ESP protein, which is responsible for the covalent bonds being formed among chorionic components and for the insolubilization of chorion structure. Utilization of PGL as inhibitor of the chorion-peroxidase reaction was first established by Mindrinos and colleagues^42^.

In the present study, follicles of stages 11, 12, 13 and 14 were carefully selected and were, next, *in vitro* developed in radio-active Robb’s medium being supplemented with [^3^H] radio-labeled L-proline, in the presence of 0.2 mM PGL, to examine the substance effect on chorionic-protein synthesis at each developmental stage. Follicles of stage 11 (s11) were treated with PGL for 30 min. Synthesis of the major chorionic proteins commences at this stage, with the s38 and s36 “early” chorionic proteins showing profiles of strong expression (Fig. 1; lane a). Developmental stage 12 (s12), which lasts 120 min, is molecularly typified by expression of all chorionic proteins belonging to the A, B and C group, and of the s19 one from the “late” proteins of group D (Fig. 1; lane b). The chorionic proteins derived from follicles of developmental stage 13 (s13) are synthesized within 60 min. Synthesis of s38, s36 and s19 is continued, while production of s18, s16 and F-protein of filaments (F) has initiated and is fully activated (Fig. 1; lane c). The “late” chorionic proteins s19, s18, s16 and s15, as well as the filament-specific component F, are all selectively synthesized during the 120 min of developmental stage 14 (s14) (Fig. 1; lane d).

Interestingly, the 0.2 mM dose of PGL does not detectably impair the follicle cell-specific protein-synthesis machinery. However, at the same time, it seems to inhibit the ESP activity, at stage 14, thus facilitating chorion-structure solubilization and protein-extract preparation. The normal electrophoretic pattern of chorionic proteins obtained, at the concentration of 0.2 mM PGL, serves as the reference point, and provides a base-line for systemically assessing the cytotoxic power of comparatively higher PGL concentrations on protein-synthesis integrity, reliability and efficacy.

### High doses of PGL impair protein-synthesis machinery in the ovarian follicle-cell layer

Follicles carrying the competence to complete the choriogenesis process *in vitro*, according to their endogenous genetic program, represent a remarkable, powerful and developmentally autonomous system that is expected to give reliable and important answers regarding the cytotoxic power of PGL on differentiated somatic, epithelial, cells of complex biological settings.

**Figure 2:**
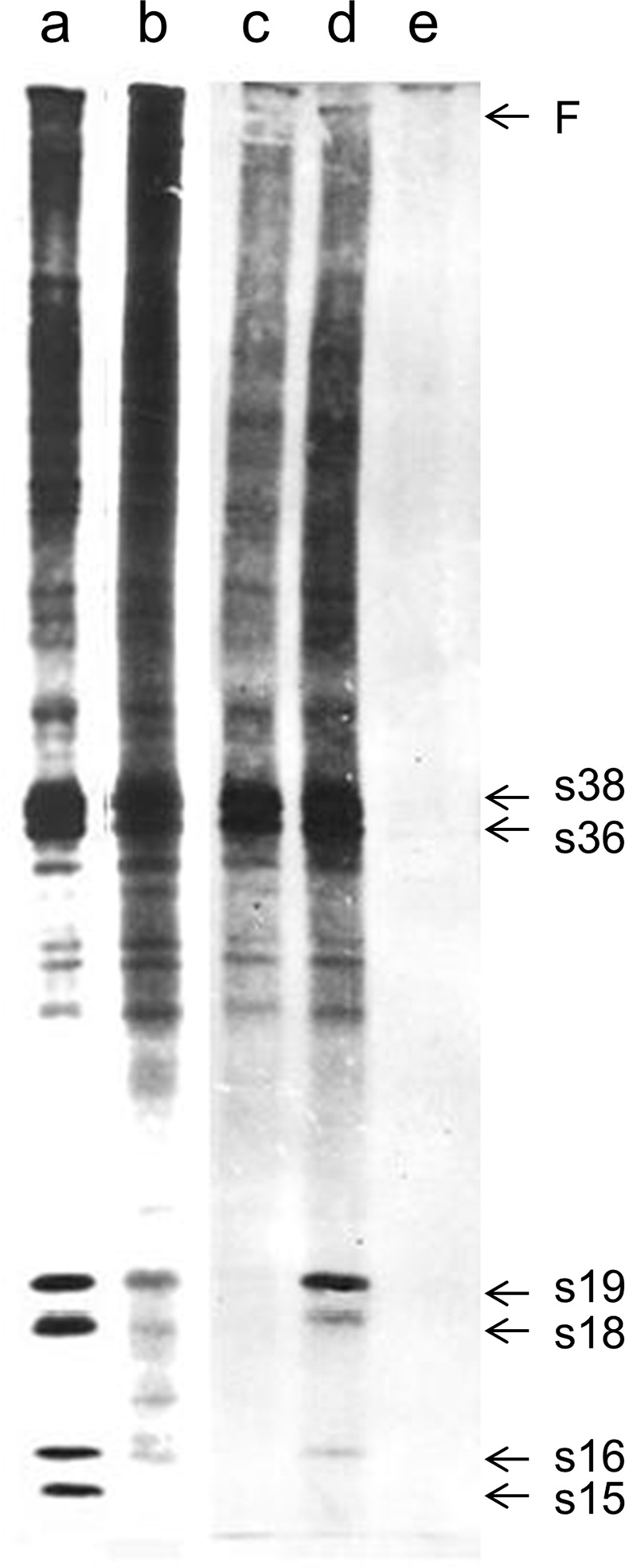
Effects of different PGL doses on chorionic protein-expression patterning. SDS-PAGE (10–15% acrylamide gradient) and autofluorography analysis of follicle cell-derived chorionic proteins, after the *in vitro* incubation of *D. melanogaster* ovarian follicles, in Robb’s culture medium, with [^3^H] radio-labeled L-proline and PGL compound. (**a**) stage-11 follicles, 1 mM PGL, 6 h incubation time. (**b**) stage-11 follicles, 10 mM PGL, 6 h incubation time. (**c**) stage-11 follicles, 20 mM PGL, 6 h incubation time. (**d**) stage-13 follicles, 20 mM PGL, 6 h incubation time. (**e**) stage-11 follicles, 40 mM PGL, 6 h incubation time. The six major chorionic proteins (s38, s36, s19, s18, s16 and s15), together with the filament-specific one (F), are indicated on the right side of the panel.

Stage-11 follicles were incubated, for 6 h, in radio-active Robb’s medium containing 1 mM PGL, and their total-protein extracts were, next, subjected to SDS-PAGE and autofluorography protocols (Fig. 2; lane a). Comparison of the obtained electrophoretic pattern with the one of Fig. 1 evidences that all chorionic proteins, belonging to the 4 (A, B, C and D) groups, are normally synthesized. Follicle’s development is physiologically completed up to stage 14, as clearly documented by production of the stage 14-specific s15 and F chorionic proteins. However, this cannot be observed upon treatment of stage-11 follicles, for 6 h, with a 10 mM PGL dose (Fig. 2; lane b). Interestingly, proteins from A, B and C group seem to be normally produced, whereas the ones of group D, s19, s18 and s16, are significantly reduced in terms of their expression levels, with the s15-protein contents being virtually undetected. Comparison of their electrophoretic profile with the one described in Fig. 1 indicates that follicle cell-dependent synthesis of low-molecular weight, “middle” and “late”, proteins is notably compromised at the initial stage 13, approximately 2–3 h after the beginning of incubation with PGL compound. Strikingly, protein synthesis of stage-11 follicles, which are being treated with 20 mM PGL, for 6 h, is severely impaired (Fig. 2; lane c). In contrast to group C, groups A and B are moderately affected, while group-D and F proteins cannot be detected. The absence of s19 dictates that protein synthesis is interrupted during stage 12, approximately 60 min post-start of PGL incubation. A different protein-resolving pattern was revealed in stage-13 follicles that were being exposed to 20 mM PGL, for 6 h (Fig. 2; lane d). The obtained electrophoretic autofluorographs suggest that all proteins belonging to groups A, B and C, and the s19 one from group D, retain their normal expression contents (as compared to the ones of Fig. 1). Nevertheless, s18 and s16 protein levels are significantly reduced, while s15 ones are presented experimentally undetected. It seems that, in response to this PGL-exposure scheme, protein synthesis is terminated in less than 60 min, within the developmental time-period of a typical stage-13 follicle. Most importantly, the 40 mM of PGL dose can induce dramatic effects on protein-synthesis efficacy of ovarian follicle cells. Protein electrophoretic profiles are characterized by hardly detected or lack of autofluorographic bands (Fig. 2; lane e), thus pointing out the protein-synthesis dramatic harm at the beginning of stage-11-follicles’ treatment with PGL high doses.

In conclusion, PGL shows a dose-dependent and progressively increasing toxicity against *Drosophila* follicular epithelium, at 10, 20 and 40 mM of its concentration, while at the 1 mM compound dose, it (PGL) can be well tolerated by the ovarian follicle cells, without downregulating the expression of examined chorionic proteins.

### PGL induces dose-specific chorion dysmorphia in *Drosophila* ovarian follicles growing *in vitro*

The extracellular protein structure of fly chorion is formed during the late developmental stages of oogenesis, in order to ensure embryo’s survival in potentially fluctuating and occasionally harsh environmental conditions. It is a highly complex structure that requires precise spatio-temporal control of cellular function at replicative (i.e. gene amplification/re-replication), transcriptional, translational and secretory level. Follicular epithelium comprises distinct cell (sub-)populations, whose different genetic programs and migration patterns harmoniously direct the morphogenesis of chorion’s specialized, critical, structures; the micropyle and the respiratory filaments (appendages). After completion of their biological duties, at the stage 14 of oogenesis, ovarian follicle cells undergo orchestrated degeneration and programmed cell death^37,43^. Hence, PGL effect on the complex, sensitive and versatile cellular system of *Drosophila* choriogenesis serves as a highly important, efficient and powerful platform to drawing reliable and accurate conclusions for the cytotoxic potency of the compound.

#### Effects of 0.2, or 1 mM of PGL dose

**Figure 3:**
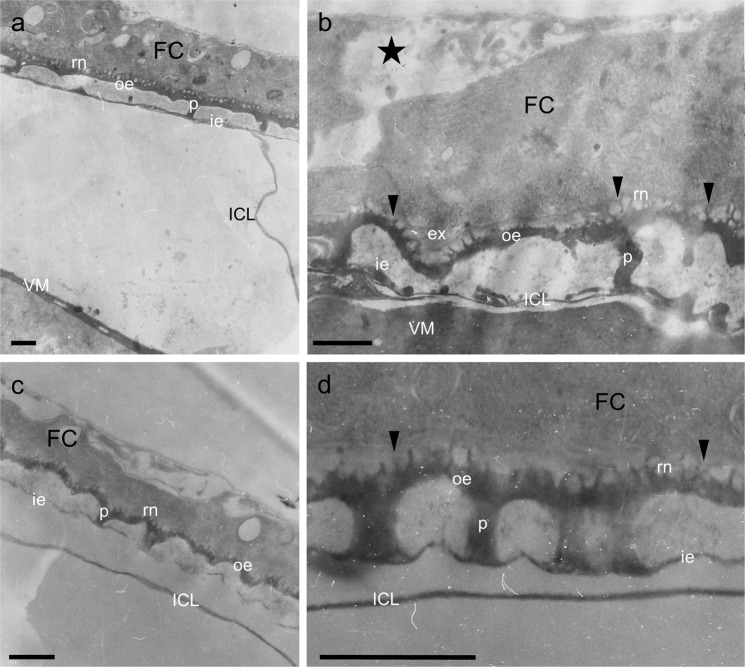
Follicle exposure to 0.2, or 1 mM PGL does not virtually disrupt chorion structure and architecture. Characteristic TEM micrographs of (thin-sectioned) ovarian follicles, after their *in vitro* growth in Robb’s medium containing 0.2, or 1 mM of PGL dose. (**a**,**b**) stage-11 follicles, 0.2 mM PGL, 6 h incubation time. (**c**,**d**) stage-11 follicles, 1 mM PGL, 6 h incubation time. ICL: inner chorionic layer; ie: inner endochorion; p: pillar(s); oe: outer endochorion; rn: roof network; FC: follicle cell; VM: vitelline membrane. Arrowheads: follicle-cell microvilli. Asterisk: cytoplasmic material being secreted from follicle cells. Scale bars: 1 μm.

PGL, at a concentration of 0.2 mM, was used in Robb’s culture medium during the *in vitro* development of stage-11 follicles, for 6 h (Fig. 3a,b). Engagement of a TEM protocol proved that the chorion structure of PGL-treated follicles is normally organized and formed, with ICL and endochorion showing absence of morphologically detectable abnormalities. Roof network is satisfactory developed and follicle-cell microvilli seem to successfully drive morphogenesis of the exochorion structure (Fig. 3b). Likewise, chorion architecture of stage-11 follicles that have been exposed to 1 mM of PGL dose, for 6 h, is presented with lack of deformities and structural abnormalities (Fig. 3c,d). Nevertheless, roof network is comparatively less developed, while several distinct microvilli can be still recognized in the follicular layer (Fig. 3d). In general, chorion biogenesis remains undisturbed, albeit a short time-delay is being observed for its completion.

#### Effects of 10 mM of PGL dose

**Figure 4:**
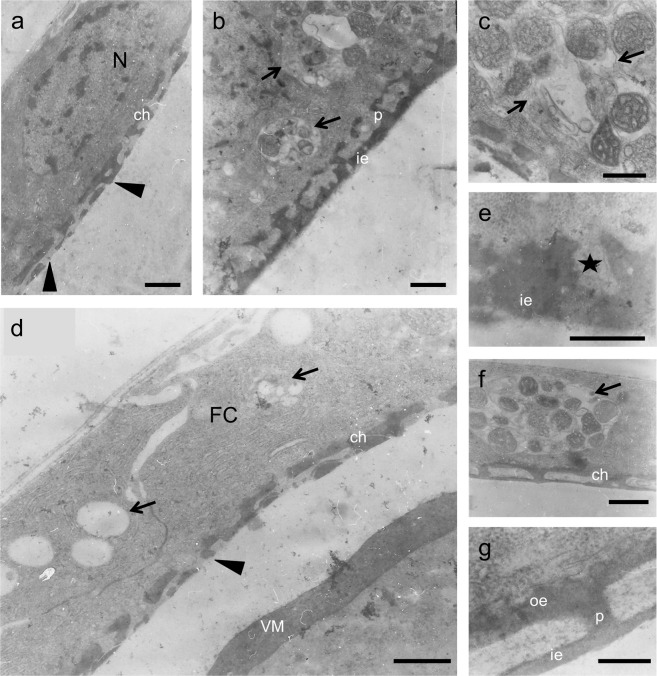
10 mM PGL induces notable pathology in chorion formation. Representative TEM micrographs of stage-11 follicles having been grown *in vitro*, for 6 h, in Robb’s medium containing 10 mM of PGL concentration. (**a**) a characteristic normal nucleus. (**a**,**b**,**d**,**f**,**g**) ICL-missing chorion. (**a**,**b**,**d**) disorganized inner endochorion and pillars in the main chorion body. (**b**–**d**,**f**) PGL-induced cytoplasmic vesicles and autophagic vacuoles. (**e**) cytoplasmic protrusions (asterisk). (**g**) abnormal structure near the posterior pole with thick floor (“ie”), pillars and roof (“oe”). Microvilli cannot be distinguished. ch: chorion; ie: inner endochorion; p: pillar(s); oe: outer endochorion; FC: follicle cell; VM: vitelline membrane; N: nucleus. Arrows: vesicles and autophagic vacuoles. Arrowheads: pathological chorion architecture. Scale bars (**a**,**b**,**d**,**f**) 1 μm; (**c**,**e**) 0.5 μm; and (**g**) 0.25 μm.

Stage-11 follicles were incubated, for 6 h, in Robb’s nutrient medium, with 10 mM of PGL concentration (Fig. 4). Remarkably, follicles could only be developed up to stage 13, with their chorion bearing structural aberrations. In the main body, ICL is unable to be morphologically recognized, while floor and pillars are notably disorganized (Fig. 4a,b,d), with the posterior pole-residing floor obtaining a thick architecture that lacks ICL (Fig. 4e–g). Regarding follicle cells, nuclei are normally organized (Fig. 4a), but in several cytoplasmic areas autophagic vacuoles seem to be formed, either detoxifying follicular layer from PGL-induced toxic material or promoting cells to undergo PGL-driven autophagic death (Fig. 4b,c,f). At the apical membrane, microvilli cannot be distinguished, while harmful components may be deposited in the cytoplasmic protrusions being formed by PGL treatment (Fig. 4g). This emerging evidence suggests that critical secretory mechanisms of PGL-affected follicle cells have been significantly compromised. PGL may derange cytoskeleton integrity and function, and may disrupt endoplasmic reticulum (ER) architecture, and secretion-process fidelity and efficacy.

#### Effects of 20 mM of PGL dose

**Figure 5:**
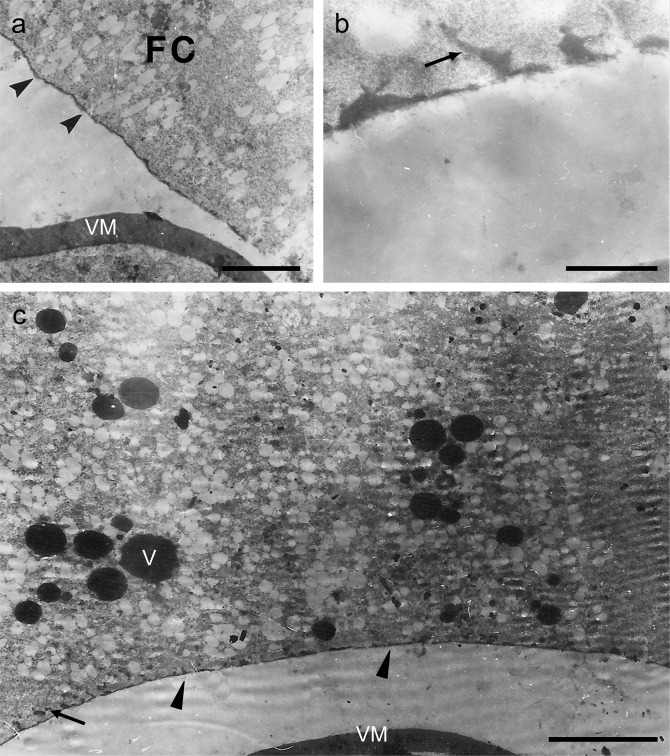
Chorion-pathology profiling, in response to follicle treatment with 20 mM PGL, for 6 h. Representative TEM micrographs from a stage-11 typical follicle, after its *in vitro* incubation in Robb’s medium, for 6 h, with 20 mM PGL. (**a**,**c**) rudimentary chorion, carrying reduced amount of chorionic material. (**b**,**c**) traces of pillars. (**a**,**c**) granular ER fragmentation-derived vesicles and cytoplasmic electron-transparent vesicles. Arrows: pillar traces. Arrowheads: rudimentary chorion. FC: follicle cell. VM: vitelline membrane. V: electron-dense vesicle (oocyte yolk-like granule). Scale bars (**a**,**b**): 1 μm; and (**c**): 5 μm.

**Figure 6:**
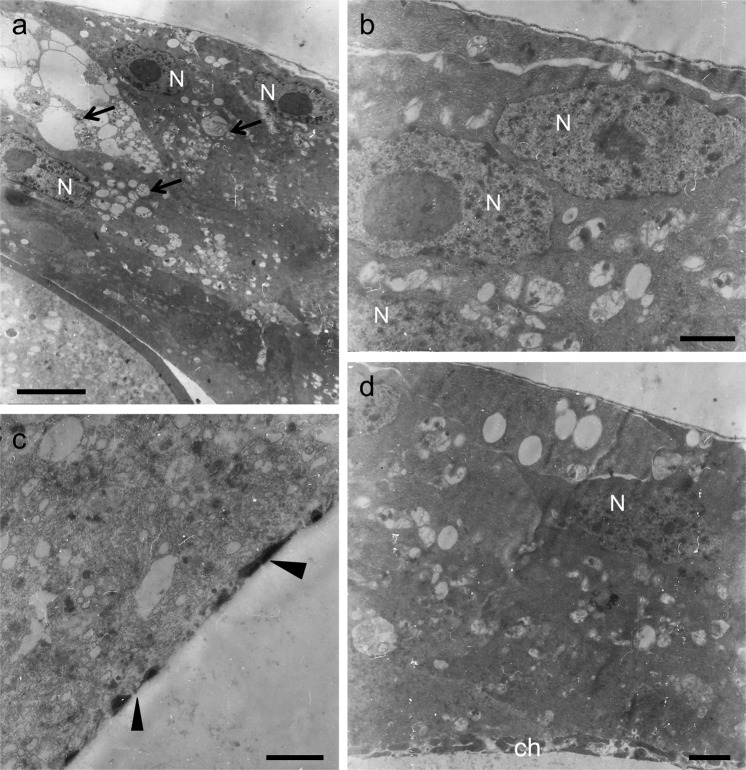
Chorion-pathology profiling, in response to follicle treatment with 20 mM PGL, for ~12 h. Characteristic TEM micrographs of stage-11 ovarian follicles, after their *in vitro* incubation, for ~12 h (overnight), in Robb’s nutrient medium containing 20 mM PGL. (**a**) a multi-layered epithelium of cells at the anterior-dorsal region of a typical affected follicle. Note the lack of deposited chorionic material. (**a**–**d**) cytoplasmic autophagic vacuoles. (**b**) syncytium-like formation being derived from plasma-membrane fusion of two (or more) neighboring follicle cells. Note the initiation of nuclear-chromatin condensation. (**c**) mono-layer cell epithelium at the main body and towards the posterior pole. Note the rudimentary chorion structure. (**d**) nuclear lobes and condensed chromatin. (**a**,**c**,**d**) electron-transparent (cytoplasmic) vesicles. Note the abnormal chorion towards the posterior pole and the absence of ICL. Arrows: autophagic vacuoles. Arrowheads: rudimentary chorion. ch: chorion. N: (follicle-cell) nucleus. Scale bars (**a**) 5 μm; and (**b**–**d**) 1 μm.

Stage-11 follicles were grown, either for 6 h or overnight (~12 h), in Robb’s culture medium containing 20 mM PGL (Figs. 5 and 6). At the PGL environment of 6 h-exposure time, chorion structure is virtually rudimentary. Excreted proteinaceous material seems to rather normally form the inner endochorion, but only architectural traces of the pillars (Fig. 5a,b). Cytoplasm is disrupted and ER is fragmented into numerous electron-transparent vesicles, look liking vacuoles (Fig. 5c). Characteristic is the accumulation of electron-dense vesicles, resembling oocyte yolk-like granules (Fig. 5c).

In relation to the ~12 h PGL-exposure setting, follicles proved capable to develop up to stage 12. At the anterior-dorsal region of an affected, typical, follicle, a multi-layered epithelium is formed, presumably due to abnormal migration of follicle-cell (sub-)populations towards the anterior pole (Fig. 6a). Cytoplasm is loaded with an abundance of autophagic vacuoles and electron-transparent vesicles (Fig. 6a,b,d). The follicular epithelium progressively decreases in thickness and formation of a syncytium-like structure is observed, as a result of follicle-cell fusions (Fig. 6b). In the main body, epithelium obtains a mono-layered architecture, and the deposited chorionic material forms rudimentary floor and traces of pillars (Fig. 6c). At the posterior pole, there is an abnormal chorionic structure that does not follow the floor-pillar-roof canonical organization pattern (Fig. 6d). In the nucleus, lobes and condensed-chromatin-elevated contents are morphologically distinguished (Fig. 6a,b,d).

Altogether, at the 20 mM of PGL concentration, follicle-cell integrity and function collapse. Cytoplasm’s pathology, being typified by abundance of vesicles and autophagic vacuoles, condensed chromatin, formation of nuclear lobes and follicle-cell fusion-mediated generation of syncytium-like assemblies, indicates that ovarian follicle cells, in response to the 20 mM of PGL dose, undergo an apoptosis- and autophagy-dependent death, with likely synergistic actions in between the two processes.

#### Effects of 40 mM of PGL dose

**Figure 7:**
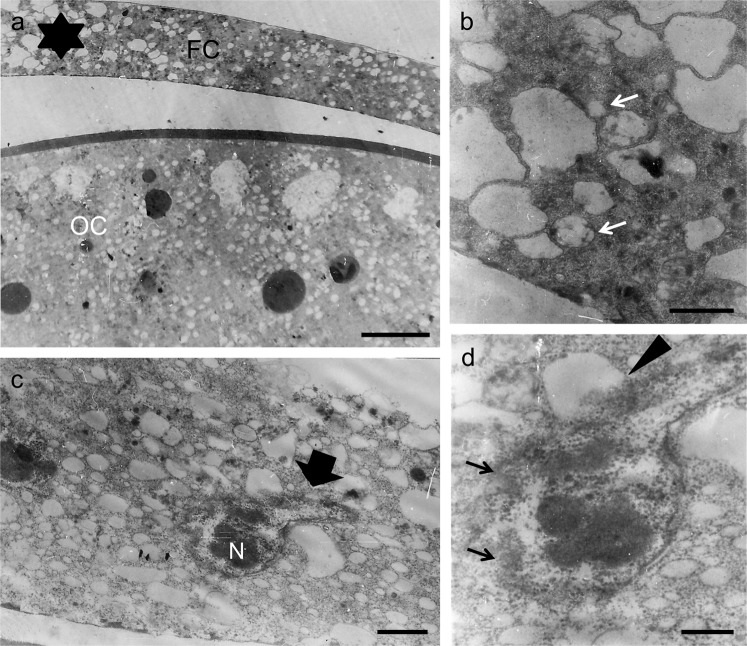
40 mM of PGL dose can cause severe follicular pathology and a “chorionless” phenotype. Representative TEM micrographs of (thin-sectioned) stage-11 follicles, after their 6 h *in vitro* growth in Robb’s nutrient medium containing 40 mM of PGL concentration. (**a**) follicle cells undergoing apoptosis (late stage) and (non-protective) autophagy. Note cytoplasm’s disintegration, and formation of numerous electron-transparent vesicles and autophagic vacuoles. (**b**) high magnification of a cytoplasmic area from “**a**” (asterisk). (**c**) nuclear protrusions, condensed chromatin and granular nucleolus. Note the fragmentation of granular ER into numerous vesicles. (**d**) high magnification of nucleus from “**c**”. Note the nucleophagy-like incident. Arrows: nuclear-envelope disruption(s). Arrowhead: disintegrated nucleus-autophagic vacuole junctions. FC: follicle cell(s). OC: oocyte. N: nucleus. Scale bars (**a**) 5 μm; (**b**,**c**) 1 μm; and (**d**) 0.5 μm.

Exposure of *Drosophila* ovarian follicles, for 6 h, to Robb’s nutrient medium supplemented with 40 mM PGL, seems to also activate apoptotic and autophagic cell-death programs. Remarkably, chorionic material is not deposited at all, while an abundance of vesicles and autophagic vacuoles can be recognized in the degenerated cytoplasm (Fig. 7a). The numerous cytoplasmic vacuoles and their -average- large size strongly imply the severe disintegration of follicular epithelium (Fig. 7b). A representative morbid nucleus carries (a) granular nucleolus, (b) disrupted nuclear envelope, (c) condensed, disorganized or/and shattered chromatin, (d) irregular lobes and (e) nucleophagy-like (nucleus-autophagic vacuole junctions) incidents (Fig. 7c,d). In conclusion, treatment of *Drosophila* ovarian follicles with 40 mM PGL results in “chorionless” phenotypes, and causes, presumably synergistic, activation of apoptotic and autophagic cell-death repertoires.

## Discussion

In our laboratory, PGL has been extensively used as an inhibitor of the chorion-hardening program characterizing *D. melanogaster* and *D. oleae* ovarian development. Our research team has previously demonstrated that addition of PGL to fly’s food at 50 mM (6.25 mg/ml) dose is unable to detectably compromise egg-production efficiency, whereas at a dose of 100 mM (12.5 mg/ml) egg-production is completely abolished and fly-males’ viability is severely shortened^56^. Therefore, at high concentrations, PGL proves to cause systemic toxicity. However, it generally belongs to those phenolic substances that are being used in veterinary and human medicine with low toxicity (EMEA/MRL/046/95-FINAL). PGL and its chemical derivatives have been previously tested for *in vivo* toxicity in several animal model systems, including *Caenorhabditis elegans* and zebrafish^57–59^. Given our previously published results and the high concentrations required for organ or systemic toxicity in either zebrafish or mice and rats, we herein considered the use of PGL *in vivo* doses of a similar millimolar (mM) scale^59,60^. Although in rats the LD_50_ values for oral PGL uptake is ~4 g/kg (bw)^10^ or ~5.2 g/kg (bw)^60^, no relevant information has been reported for humans. Importantly, PGL has been approved by several countries around the world as an effective drug against irritable bowel syndrome^61,62^. Nevertheless, its safe use has not been conclusively documented, yet. The 80 mg of PGL, three times daily oral administration^4^, for treatment of irritable-bowel-syndrome symptoms, together with the intravenous injection of 200 mg in 20 ml of saline to treat acute renal colic^63,64^ seem as PGL safe-dose range for humans. This dosage of ~1–3 mg/kg (bw) is very small in relation to the respective one used in experimental animal-model systems.

Specifically, oral administration of PGL in diabetic rats, at a concentration of 100, 200 or 250 mg/kg (bw), improved the levels of glucose, proteins and creatinine, by exerting protective effects against diabetes-mellitus complications^10^. In mice, treatment with PGL, at 100 mg/kg (bw) dose, reduces glucose tolerance by partially inhibiting the phospho-enol pyruvate carboxy-kinase (PEPCK) and glucose-6-phosphatase (G6Pase) enzymes in the liver, and influencing the AMP-activated protein kinase α (AMPKα) signaling pathway^12^. Low doses of PGL oral administration (1–16 mg/kg) have been causatively associated with anti-anxiety effects in mice, while its high doses even up to 300 mg/kg (bw) could be well tolerated by the experimental animals^13^. In another pharmacological setting, PGL intra-peritoneal administration in four doses (25 mg/kg), every other day, in mice, reduced the metastatic ability of breast cancer cells^26^.

Given the dose-dependent ability of PGL to exert either beneficial or harmful systemic effects in model biological environments, the critical issue herein being raised is to determine the maximum concentration of PGL compound that does not adversely affect the structural and functional integrity of differentiated somatic cells and tissues. *Drosophila* choriogenesis has herein proved to serve as an ideal biological system for the assessment of compound cytotoxicity, with emphasis on PGL detrimental power. The oocyte is surrounded by approximately 650 differentiated, epithelial (somatic), follicle cells, which in 6 h can form the chorion, an extracellular protein structure with a multi-functional role. Hence, the thorough examination of PGL impact on *Drosophila* choriogenesis, at biochemical, morphological and developmental level, provides strong evidence for its high dose-specific toxicity in complex, meta-mitotic, multi-cellular systems.

After the *in vitro* growth of ovarian follicles in culture medium containing 0.2 mM (25.2 μg/ml) of PGL dose, chorionic proteins are normally synthesized at each developmental stage and chorion is formed without detectable alterations in its structure. PGL, at a concentration of 1 mM (126 μg/ml), seems to be well tolerated by treated follicles and does not cause abnormalities in chorion’s biogenesis. The individual chorion layers, ICL, floor, pillars and roof, are formed in approximately 6 h, as expected, and the chorionic proteins of stage 11 to 14 are normally expressed, following their canonical developmental-regulation patterning. Altogether, it seems that the PGL concentration of 126 μg/ml is unable to cause measurable toxicity in somatic, epithelial, follicle cells, during *Drosophila* choriogenesis. Remarkably, in LLC-PK1 renal epithelial cells, PGL shows the greatest protective effect against induced oxidative stress at a concentration of 100 μg/ml (0.8 mM). Furthermore, exposure of WI-38 fibroblast cells to 25–100 μg/ml PGL increases their viability during H_2_O_2_-induced premature cell aging^20^.

However, other observations indicate the low toxicity of PGL at doses higher than 10 μg/ml. SH-SY5Y (neuroblastoma) cells experience maximum anti-oxidant and cyto-protective outcomes at a compound concentration of 10 μg/ml (0.08 mM), whereas 40 μg/ml (0.32 mM) of PGL significantly reduces cell viability^18^. Likewise, at doses of 20, 30, 40 and 50 μg/ml, PGL progressively decreases viability of HT-22 (immortalized mouse hippocampal) cells by 5, 10, 20 and 25%, respectively, with the 10 μg/ml concentration presenting an absence of effect^21^. The 50 μg/ml (0.4 mM) PGL proved ineffective on rat small-intestine epithelial cells (IEC-6), but could cause 60% reduction in the viability of HT-29 colon-cancer cells. In general, cancer cells, or immortalized cell lines, seem to differentially respond to PGL actions, as compared to normal cells, manifesting an increased PGL sensitivity^30^. This finding opens a new therapeutic window for PGL as a promising anti-cancer agent in the future.

We have herein proved that PGL at concentrations higher than 1 mM (126 μg/ml) can exert cellular toxicity in the *Drosophila* choriogenesis model process. 10 mM (1.26 mg/ml) of the compound severely impairs protein-synthesis integrity and efficacy, at stage 13 of follicle development, with chorion structure presenting a serious pathology, being tightly associated with chorionic-protein-synthesis downregulation. Disorganization of actin-cytoskeleton network and loss of microvilli, as previously reported for follicle cells undergoing apoptosis^37^, may also contribute to the PGL-induced biogenesis of dysmorphic chorion. Similarly, in ovarian follicles being treated with the apoptotic inducer staurosporine, a completely disorganized actin network was observed^65^. In the PGL-affected follicular epithelium, a number of autophagic vacuoles were observed in the cytoplasm, strongly suggesting the follicle cell-specific activation of autophagic cell death, a program previously described to operate during late-oogenesis completion process^43^. It seems that apoptotic and autophagic machineries successfully synergize to drive impaired follicle cells to death. This may be also supported by mechanisms of PGL-mediated ER-stress activation, as previously shown for other cellular settings^66,67^. Remarkably, at the PGL concentration of 20 mM (2.5 mg/ml), cellular physiology of developing follicles was severely damaged by multiple ways. Follicle cell-specific protein synthesis was interrupted at the choriogenic stage 12. In the cytoplasm, numerous electron-transparent and electron-dense vesicles (oocyte yolk-like granules) were accumulated, likely indicating the failure of follicle cells to embody protein material into the developing chorion structure. Strikingly, in follicle cells lacking the chorionic protein s36, heavy loads of electron-dense vesicles were detected^68^. The ~12 h (overnight) exposure of stage-11 follicles to 20 mM PGL caused elevation of nuclear chromatin-condensed contents, and formation of multi-layered epithelium and syncytium-like structures, presumably derived from cell-fusion incidents, being tightly associated with plasma-membrane pathologies, during follicle cell-population migration courses^35,69^. Altogether, PGL is presented as highly competent to severely harm the secretion process and to notably disrupt the granular ER compartment of follicle-cell layer. Accumulation of condensed chromatin and cytoplasmic autophagosomes reflect the synergistic activities of apoptotic and autophagic cell-death programs, as reported before in other biological environments^43^.

The highly increased PGL dose of 40 mM (5 mg/ml) resulted in complete inhibition of protein synthesis and massive fragmentation of granular ER into numerous vesicles of varied sizes. Nucleus becomes apoptotic, carrying condensed chromatin, granular nucleolus, nuclear-envelope disruptions and irregular protrusions. It seems to be also subjected to nucleophagy-dependent elimination, as morphologically dictated by the identification of junctions in between a typical degenerated (apoptotic) nucleus and an autophagic vacuole. In the disintegrating cytoplasm, assembled autophagosomes and large vacuoles are destined for engulfing PGL-directed apoptotic remnants of follicular epithelium. Interestingly, programmed cell death of follicle cells can be observed during stage 14B of ovarian-follicle development and upon completion of chorion-structure synthesis^37,43^.

Importantly, PGL seems to significantly affect calcium channels and cytochromes, thus promoting activation of apoptosis via engagement of the ER-stress pathway^66,70–72^. Since cell fusion can be induced by disorders in calcium (Ca^2+^) homeostasis or/and G-protein activity^73^, while it can be also enhanced by hypoxia^74^, a PGL-driven deregulation and imbalance of Ca^2+^ contents may compel adjacent follicle cells to fuse their plasma membranes, thus leading to a syncytium-like structure. An increase in intracellular Ca^2+^ concentration has proved insufficient to induce apoptosis in endothelial cells, whereas a decrease in the levels of ER-residing Ca^2+^ is proficient in triggering off apoptosis, independently of caspase-12 activation^75^. Hence, it must be the Ca^2+^ release from ER-disintegrating compartment that mediates apoptosis of follicle cells upon their exposure to high PGL concentrations. Providing the PGL-orchestrated impairment of follicle cell-secretion machinery, proteins may reasonably accumulate in ER cisternae leading to ER-stress responses that activate apoptosis. In accordance, Bortezomib-mediated inhibition of *Drosophila* ovarian-follicle proteasome is mechanistically coupled to induction of ER-stress signaling mechanism, and to synergistic activation of apoptosis and autophagy^67^.

*In toto*, PGL, at doses up to 1 mM (126 μg/ml), does not harm the structure, organization, architecture and function of differentiated somatic, epithelial, follicle cells, during *Drosophila* choriogenesis model process. Thereby, this concentration may serve as a critical threshold for non-toxic and safe PGL administration in complex biological systems requiring some kind of beneficial treatment. Non-toxic PGL doses are expected to open new therapeutic windows, in the form of combined targeted therapies, for a number of catastrophic diseases, including human malignancies.
